# In an unhealthy food system, what role should SNAP play?

**DOI:** 10.1371/journal.pmed.1002662

**Published:** 2018-10-02

**Authors:** Hilary Kessler Seligman, Sanjay Basu

**Affiliations:** 1 Department of Medicine, University of California San Francisco, San Francisco, California, United States of America; 2 Department of Medicine, Stanford University, Palo Alto, California, United States of America

## Abstract

In a Perspective, Hilary Seligman and Sanjay Basu discuss future scenarios of food assistance programs to improve population health in a changing political environment.

It is no longer controversial to say that the United States food system does not support a healthy diet. Junk food is extraordinarily palatable and virtually omnipresent; its advertising is pervasive; many Americans do not live within convenient distance of a grocery store stocking healthy alternatives; and healthier foods are typically perceived as costlier. In this environment, the Supplemental Nutrition Assistance Program (SNAP) provides 42 million low-income people with financial assistance to purchase food. Most SNAP recipients, because they tend to live in lower-income communities, are exposed to the worst of the US food system: more unhealthy food marketing through traditional and social media, more unhealthy foods in the stores where they regularly shop, and fewer healthy foods that are financially within reach. Although SNAP benefits are intended to provide low-income families with sufficient food-purchasing power to obtain a nutritious diet, there is broad consensus that current benefits are insufficient [1]. The US food system is in urgent need of policies and programs that support and facilitate better dietary habits.

In a microsimulation modeling study in this issue of *PLOS Medicine*, Dariush Mozaffarian and colleagues examine one such policy: leveraging SNAP to incentivize healthy food purchases and disincentivize unhealthy food purchases [2]. Their model estimates that a 30% incentive on fruit and vegetable purchases would avert almost 12,000 cardiovascular deaths over the next 20 years, whereas combining a fruit and vegetable incentive with a restriction on purchases of sugar-sweetened beverages or combining a fruit and vegetable incentive with a restriction on a broad swath of unhealthy foods would avert even more cardiovascular deaths (40,420 and 48,088, respectively). Substantial cost savings are anticipated by this model.

The authors estimate the effect size of the proposed fruit and vegetable incentive from outcomes of a prior randomized trial [3], which strengthens confidence in this portion of their results (the effect sizes of a sugar-sweetened beverage purchasing restriction are more uncertain). The modeling is nicely validated in the components of the simulation related to cardiovascular disease risk estimation. However, as with all modeling studies, the authors needed to make assumptions. They did not explicitly simulate substitution between food groups (e.g., from sugar-sweetened beverages to 100% fruit juice); in the restriction scenarios, there is limited compensation for use of non-SNAP dollars to buy foods not allowed with SNAP dollars; and they assume that cardiac risk reductions from temporary improvements in diet will persist in perpetuity, even if a person only receives SNAP for a brief period. Despite these assumptions, their findings align with prior models that made alternative assumptions, similarly showing that modifications to SNAP can positively influence dietary intake among low-income people and generate substantial health benefits and cost savings [4,5].

Nevertheless, a large question looms over the findings: in the current political environment, how should public health practitioners and SNAP policymakers proceed with these results?

## The root cause of diet-related disease is an unhealthy food system

We contend that any SNAP policy changes must be introduced and framed as part of a broader effort to address perversities of the US food system. Public health researchers must avoid promoting the idea that SNAP as a program or SNAP recipients themselves are a cause of the US epidemic of diet-sensitive chronic disease and health disparities, when the root cause is an unhealthy food system. Although diets of SNAP recipients are poorer than diets of non-SNAP recipients, differences are relatively small and may be attributable to unmeasured confounders. For example, point-of-sale data suggest that there are few major differences in expenditure patterns of SNAP and non-SNAP households, with about 40 cents of every food dollar spent on basic items (meat, fruits, vegetables, milk, eggs, and bread) and 20 cents spent on junk food and sugar-sweetened beverages [6]. Sugar-sweetened beverages rank first in expenditures for SNAP households but second (just after milk) for non-SNAP households. Although added-sugar acquisitions are higher among SNAP participants than similar nonparticipants (31 tsp-eq versus 23 tsp-eq daily), overall diet quality as measured by the Healthy Eating Index is similar [7]. Studies correlating SNAP enrollment to obesity have been heavily publicized, but associations between SNAP and obesity disappear when adequately adjusting for unmeasured confounders [8]. In fact, many Americans—not just SNAP recipients—do not make healthy food choices in the current food system [9].

Public health research (including our own) on leveraging SNAP to improve dietary intake assumes that federal policymakers are interested in creating opportunities for healthier dietary intake and reducing health disparities. However, some policymakers have reframed this public health research to identify SNAP or SNAP recipients as the problem, playing into a broader agenda set on eliminating the social safety net and stigmatizing the poor.

In this climate, research can become misrepresented in the media or by politicians to suggest that SNAP as a program, or SNAP participants as a population, is to blame for unhealthy dietary practices that are draining the US healthcare system.

This misrepresentation risks inadvertently contributing to program defunding or eligibility restrictions, which may neutralize any public health benefits or even result in perversely worse health outcomes.

## SNAP has far-reaching benefits

Policy changes must not negate SNAP’s far-reaching benefits. SNAP reduces food insecurity rates by 20%–30% [10]. Children born to mothers receiving SNAP are healthier at birth and less likely to develop the metabolic syndrome in adulthood compared with similar children born to mothers not receiving SNAP. Children receiving SNAP are also more likely to reach their full educational and cognitive potential and more likely to become economically self-sufficient [11]. Among adults, SNAP is associated with lower risk of chronic disease and lower healthcare expenditures [12]. SNAP has been associated with reduced recidivism among newly released prisoners [13]. SNAP dollars are also immediately reinvested into the US economy; the US Department of Agriculture (USDA) estimates that every $1 billion in SNAP benefits results in $1.8 billion in economic activity and creates 8,900–17,900 full-time-equivalent jobs [14]. Finally, SNAP is by design responsive to changes in the US economy, supporting more households during economic downturns and fewer households during economic expansions. Hence, any policy modifications to SNAP must be rigorously studied not only for their impact on dietary intake but also for their impact on these important collateral benefits.

## Opportunities for reform

The large health benefits from modifying dietary intake through SNAP predicted in Mozaffarian and colleagues’ article indicate a need to explore the importance of programs similar to SNAP across all levels of government. SNAP is often the focus of public health research because it is a large, federal program, but this also makes SNAP a more difficult target for effective reform than local and state initiatives, which have often been subject to less legislative gridlock. Congress renegotiates the Farm Bill that funds SNAP every 5 years, and it is extremely unlikely that the bill currently under negotiation will substantially change allowable SNAP purchases (outside of pilot projects). Major changes are unlikely to be substantively debated again until about 2023. Meanwhile, numerous state and local policies and programs to support healthier dietary intake, particularly in low-income communities, are already being discussed, implemented, and expanded. These programs, including vouchers to support fruit and vegetable purchases, healthy food procurement policies, and workplace bans on sugar-sweetened beverage sales, have generally been underresearched.

Discussing SNAP research requires grappling with the political and ethical tensions that arise when a social safety net program primarily created to address poverty is leveraged to address public health harms resulting from unjust and inequitable food systems. Lessons from studies such as this one—that systems supporting healthier dietary intake, particularly in low-income communities, can have profound public health impacts—provide good reason to support vibrant research efforts beyond SNAP alone.

## Abbreviations

SNAP: Supplemental Nutrition Assistance Program
USDA: United States Department of Agriculture

## References

[1] IOM (Institute of Medicine) and NRC (National Research Council). Supplemental Nutrition Assistance Program: Examining the evidence to define benefit adequacy. Washington, DC: 2013. 10.3945/an.113.003822 PMC3941827 23858096

[2] Mozaffarian D, Liu J., Sy S, Huang Y, Rehm C., Lee Y, et al Cost-effectiveness of financial incentives and disincentives for improving food purchases and health through the U.S. Supplemental Nutrition Assistance Program (SNAP): A microsimulation study. PLoS Med. 2018;15(10): e1002661 10.1371/journal.pmed.1002661 PMC6168180 30278053

[3] Olsho LE, Klerman JA, Wilde PE, Bartlett S. Financial incentives increase fruit and vegetable intake among Supplemental Nutrition Assistance Program participants: a randomized controlled trial of the USDA Healthy Incentives Pilot. Am J Clin Nutr. 2016;104(2):423–35. Epub 2016/06/24. 10.3945/ajcn.115.129320 . 27334234

[4] Basu S, Seligman H, Bhattacharya J. Nutritional policy changes in the supplemental nutrition assistance program: a microsimulation and cost-effectiveness analysis. Med Decis Making. 2013;33(7):937–48. Epub 2013/07/03. 10.1177/0272989X13493971 . 23811757

[5] Basu S, Seligman HK, Gardner C, Bhattacharya J. Ending SNAP subsidies for sugar-sweetened beverages could reduce obesity and type 2 diabetes. Health aff. 2014;33(6):1032–9. Epub 2014/06/04. 10.1377/hlthaff.2013.1246 . 24889953 PMC7421782

[6] Garasky S, Mbwana K, Romualdo A, Tenaglio A, Roy M. Foods Typically Purchased by SNAP Households IMPAQ International, LLC for USDA Food and Nutrition Service, 2016.

[7] Basu S, Wimer C, Seligman H. Moderation of the Relation of County-Level Cost of Living to Nutrition by the Supplemental Nutrition Assistance Program. Am J Public Health. 2016;106(11):2064–70. Epub 2016/09/16. 10.2105/AJPH.2016.303439 ; PubMed Central PMCID: PMC5055794. 27631742 PMC5055794

[8] Rigdon J, Berkowitz SA, Seligman HK, Basu S. Re-evaluating associations between the Supplemental Nutrition Assistance Program participation and body mass index in the context of unmeasured confounders. Soc Sci Med. 2017;192:112–24. Epub 2017/10/02. 10.1016/j.socscimed.2017.09.020 ; PubMed Central PMCID: PMC5815398. 28965002 PMC5815398

[9] Rehm CD, Peñalvo JL, Afshin A, Mozaffarian D. Dietary intake among us adults, 1999–2012. JAMA. 2016;315(23):2542–53. 10.1001/jama.2016.7491 27327801 PMC6287627

[10] Ratcliffe C, McKernan SM, Zhang S. How Much Does the Supplemental Nutrition Assistance Program Reduce Food Insecurity? Am J Agric Econ. 2011;93(4):1082–98. Epub 2011/01/01. 10.1093/ajae/aar026 ; PubMed Central PMCID: PMC4154696. 25197100 PMC4154696

[11] Hoynes H, Schanzenbach DW, Almond D. Long-Run Impacts of Childhood Access to the Safety Net. Am Econ Rev. 2016;106(4):903–34. 10.1257/aer.20130375

[12] Berkowitz SA, Seligman HK, Rigdon J, Meigs JB, Basu S. Supplemental Nutrition Assistance Program (SNAP) Participation and Health Care Expenditures Among Low-Income Adults. JAMA Intern Med. 2017 Epub 2017/10/04. 10.1001/jamainternmed.2017.4841 . 28973507 PMC5710268

[13] Yang CS. Does Public Assistance Reduce Recidivism? Amer Econ Rev. 2017;107(5):551–55. 10.1257/aer.p20171001 29558067

[14] Hanson K. The Food Assistance National Input-Output Multiplier (FANIOM) Model and Stimulus Effects of SNAP U.S. Dept. of Agriculture ERS; 2010 ERR-103.

